# Electrocardiogram sampling frequency for the optimal performance of complexity analysis and machine learning models: Discrimination between patients with and without paroxysmal atrial fibrillation using sinus rhythm electrocardiograms

**DOI:** 10.1016/j.hroo.2024.11.002

**Published:** 2024-11-08

**Authors:** Steven Creasy, Vadim Alexeenko, Gregory Y.H. Lip, Gary Tse, Philip J. Aston, Kamalan Jeevaratnam

**Affiliations:** 1 Department of Comparative Biomedical Sciences, School of Veterinary Medicine, University of Surrey, Guildford, United Kingdom; 2 Department of Mathematics, University of Surrey, Guildford, United Kingdom; 3 Liverpool Centre for Cardiovascular Science, University of Liverpool, Liverpool John Moores University and Liverpool Heart & Chest Hospital, Liverpool, United Kingdom; 4 Danish Center for Health Services Research, Department of Clinical Medicine, Aalborg University, Aalborg, Denmark; 5 Cardiovascular Analytics Group, PowerHealth Limited, Hong Kong, China; 6 Tianjin Key Laboratory of Ionic-Molecular Function of Cardiovascular Disease, Department of Cardiology, Tianjin Institute of Cardiology, Second Hospital of Tianjin Medical University, Tianjin, China; 7 School of Nursing and Health Studies, Hong Kong Metropolitan University, Hong Kong, China

**Keywords:** Atrial fibrillation, Complexity analysis, Machine learning, Prediction, Electrocardiogram

## Abstract

**Background:**

The current clinical practice to diagnose atrial fibrillation (AF) requires repeated episodic monitoring and significantly underperform in their ability to detect AF episodes.

**Objective:**

There is therefore potential for artificial intelligence–based methods to assist in the detection of AF. Better understanding of the optimal parameters for this detection can potentially improve the sensitivity for detecting AF.

**Methods:**

Ten-second, 12-lead electrocardiogram signals were analyzed using complexity algorithms combined with machine learning techniques to predict patients who had a previously detected AF episode but had since returned to normal sinus rhythm. An investigation was performed into the impact of the sampling frequency of the electrocardiogram signal on the accuracy of the machine learning models used.

**Results:**

Using a single complexity algorithm showed a peak accuracy of 0.69 when using signals sampled at 125 Hz. In particular, it was noted that improved accuracy occurred when using lead V_6_ compared with other available leads.

**Conclusion:**

Based on these results, there is potential for 12-lead electrocardiogram signals to be recorded at 125 Hz as standard and used in conjunction with complexity analysis to aid in the detection of patients with AF.

**Key Findings**

▪ Paroxysmal atrial fibrillation is a difficult condition to diagnose, but the use of machine learning can improve detection rates.
▪ Complexity analysis has previously been used in equine work to detect paroxysmal atrial fibrillation, but this is the first time it has been used in human patients.
▪ Sampling frequencies common in electrocardiogram devices were tested to investigate and formalize the process of using complexity analysis in the future.
▪ These methods have achieved an accuracy of ∼70%, a vast improvement on the current long-term monitoring methods.

## Introduction

Atrial fibrillation (AF) is a common and underdiagnosed heart condition associated with increased risks of stroke, heart failure, dementia, and mortality.^1^^,^^2^ AF affects 5% of the population older than 69 years and 8% older than 80 years.^3^^,^^4^ The risk of stroke in patients with AF is at least 5 times higher than that in healthy individuals.^5^ The Framingham Heart Study data show that patients with AF are at twice the risk of mortality compared with those without AF.^6^

The European Society of Cardiology guidelines^6^ define AF as “[a] supraventricular tachyarrhythmia with uncoordinated atrial electrical activation and consequently ineffective atrial contraction.” AF has electrocardiographic (ECG) characteristics of irregularly irregular R-R intervals, absence of distinct repeating P waves, and irregular atrial activations. Clinical AF requires an episode matching these criteria lasting at least 30 seconds. Approximately 25%–62% of AF cases present themselves as paroxysmal atrial fibrillation (PAF)^7^; these are recurrent episodes of AF alternating with sinus rhythm (SR) that terminates spontaneously or with intervention within 7 days.^6^

Screening for PAF can be challenging because of the low diagnostic yield of a single ECG due to the low percentage of time a patient is in AF (AF burden). Long-term monitoring is cumbersome and uncomfortable for patients.^8^ Undergoing daily 10-second ECG recordings for 2 weeks has a sensitivity of <5%.^9^ As the current clinically accepted requirement for a firm diagnosis requires an episode to be witnessed, long-term monitoring is necessary. With a projected increase in PAF burden because of an aging population,^10^^,^^11^ high-yield, cost-effective screening methods are needed.^12^

Previous analyses have focused on the identification of AF, both with the intention of detecting when an AF episode occurs and for diagnosis after reversion to SR.^13^^,^^14^ Because of the paroxysmal nature of this condition, previous work to detect PAF from SR recordings has required the use of artificial intelligence (AI) to help identify minute changes in ECG signals between those with and without PAF. A cardiologist may not be able to discern a difference between a healthy patient and a patient with PAF in SR, but AI models have the advantage of training and comparing against thousands of previous ECGs to make a prediction of diagnosis. These models have the advantage that they can include many different metrics beyond the raw signal to influence the diagnosis, including abstract methods such as complexity score.^15^

The sampling frequency of the ECGs provided to the AI models, both those using the full signal and those using metrics such as complexity score, would affect the accuracy. Yet, no prior work has been done regarding the impact of sampling frequency on the diagnosis of PAF. Therefore, we investigated the impact of sampling frequency on the diagnosis of PAF using an ECG in SR after an episode has occurred.

## Methods

### Data set choice

The main considerations when deciding the data set were to ensure that the data were of high quality with appropriate accompanying metadata. Because of the increased prevalence of PAF in older patients,^16^ it was important to know the ages of patients associated with each record to allow a fair comparison between cases and controls. For this study cases were considered to be patients with confirmed PAF and controls were patients with no confirmed PAF. PTB-XL^17^, ^18^, ^19^ was selected as the database because of the high number of good-quality strips with large amounts of metadata. The length of each strip of our study is short (10 seconds) but in keeping with clinical practice. Short ECG strips show good correlation with long (5-minute) ECG strips for heart rate variability analysis.^20^ It was therefore agreed that this data set would closely mimic those expected from a clinical and primary care setting.

The PTB-XL database contains 21,799 records from 18,869 patients, including a range of cardiac conditions. Each record is 10 seconds long and sampled at 500 Hz, with metadata including age, sex, and other observed conditions. Some records also include height and weight; however with >60% of missing data, these were omitted from our data set. Wagner et al^18^ outlined the different diagnostic and rhythm changes that are recorded. The rhythm statement was used to determine those patients with PAF by choosing patients with AF and SR records; however, it should be noted that there was no mention of the type of AF experienced by the patient and so some records may correspond to persistent AF.

### Data set breakdown

In line with the definition of PAF and the intent of using SR signals, records were chosen for patients with a confirmed AF record and at least 1 SR record recorded after the known AF record. All such records were used to maximize the number of cases. All records were classified into different age categories of <20, 21–30 with 10-year increments up to 90, and >90 years.

To create a balanced data set, an equal number of control patients exhibiting SR was selected where sex and age were matched. It was discovered that there were not enough patients to exactly match age and hence the above age ranges were selected. Similarly, no further matching was attempted (comorbidities etc) to avoid the need for matching that was not possible. SR records with no comorbidities were selected to avoid needing further matching.

This resulted in 364 records, split equally into 182 cases and 182 controls. Table 1 presents the distribution of these patients across age groups. After matching, this data set is skewed more toward male patients, with 222 male patients to 144 female patients. PAF was more frequently observed in men in terms of age-adjusted incidence, but similar numbers were observed for both sexes for the whole cohort because of higher life expectancy in female patients. After matching, there are a higher number of male patients overall, but the highest age ranges contain a higher proportion of female patients.

**Table 1:** Breakdown of the age groups within the training data set split into PAF and control groups including the number of male and female patients within each set

Age group (y)	Total patients	Male controls	Female controls	Male patients with PAF	Female patients with PAF
<20	0	0	0	0	0
20–29	4	1	1	1	1
30–39	12	3	3	3	3
40–49	14	6	1	6	1
50–59	74	26	11	26	11
60–69	90	39	6	39	6
70–79	86	23	20	23	20
80–89	82	12	29	12	29
≥90	2	1	0	1	0
*Total*	*364*	*111*	*71*	*111*	*71*

PAF = paroxysmal atrial fibrillation.

### Complexity methods

Complexity analysis consists of 2 distinct steps: coarse graining and complexity algorithms. First, the coarse graining method converts the ECG signal into a symbolic string as the complexity algorithms cannot work with the raw ECG signals. For this study, binary strings were used and so coarse graining techniques converted the signal to a string of 1’s and 0’s. Second, the chosen complexity algorithm assigns a complexity score to the binary string via pattern recognition methods.

### Coarse graining

We considered 4 coarse graining methods, the first of which was threshold crossing (TC). This method assigns 0 to all points that are equal to or below a predefined threshold and 1 to all points above the threshold. This threshold can be taken in any way, and future work may aim to optimize this method by investigating changes in this threshold, but here the threshold was chosen as the median value of the signal. This is a quick and computationally inexpensive way to parse the signal, as it can quite easily pick up large features such as the R-wave peak. However, this technique is susceptible to baseline wander or noise,^21^ which affects the analysis negatively, as the complexity algorithms detect changes in noise rather than changes in signal morphology.

K-means (KM) works in a way similar to TC, but the threshold is chosen using a different algorithm. A small positive and negative deflection from the mean value is chosen arbitrarily to give 2 centroids, and the distance between each point and both centroids is found. Each point that is closer to centroid 1 is assigned to 0 and points closer to centroid 2 are assigned to 1. The mean of all points closest to each centroid is then taken to update each centroid, and the distance of each point to the centroids is recalculated. Any points that are now closer to the opposite centroid are reassigned, and the mean distance of all points to the nearest centroid is taken. If the difference between the mean distance at this step and the mean distance at the previous step is below a prescribed tolerance, then the method ends; otherwise, the centroids are updated and the method iterates until the tolerance is achieved or the number of iterations reaches the length of the signal.^22^

The third coarse graining method was feature detection (FD). For this, all points are assigned to 0 apart from certain features such as the P-, R-, and T-wave peaks, which are assigned 1. As many or as few features can be selected as needed; however, for this article, the position of the P-, Q-, R-, S-, and T-wave peaks was used to retain the largest amount of information available. Detection of some peaks such as the P- and T-wave peaks can be challenging,^23^ but by determining the position of more features, it retains more information.

The final method, beat detection (BD), can be considered as a subset of FD. This method assigns 0 at all points, but 1 is assigned only at the R-wave peaks, thus highlighting only the heartbeats from the signal. This method has the advantage that noise has a much smaller impact, but a large amount of information about the signal is lost. This is the standard method used in most medical hardware.

For BD and FD, a MATLAB toolbox called ECGdeli^24^ was used to locate the features of the signal. These points were used to assign the positions of each one within the relevant signal.

Changing the sampling frequency will change the binary string generated for each signal. While methods such as BD and FD do not see a change in the relative positions of the selected features, the number of zeros interspacing them is different and affects the complexity scores given by the following methods.

## Complexity algorithms

Once a binary string is generated, it is passed to the complexity algorithms to estimate its level of complexity. In this study, LZ76, LZ78,^25^^,^^26^ and Titchener^27^ methods are used. The historical aspects of their development can be found in Online Supplemental Appendix. After their application, complexity values of the signals are then compared. However, a longer signal will generally have a higher complexity value and so normalization for strings of different lengths (corresponding to different sampling frequencies) is performed to allow easy comparison between sampling frequencies. Normalization was performed for all complexity values by dividing by n/log_2_(n), where n is the string length. This ensures that every string has a complexity between zero and one as this has been shown to be the upper bound for LZ76 and LZ78.^28^^,^^29^ For Titchener, this bound was shown to be true in some cases but not all.^30^ While this remains an open problem,^31^ we will still use this to normalize our complexity value.

### Resampling and optimization

The data set was originally sampled at 500 Hz, and we investigated the impact of sampling frequencies of 125, 250, and 375 Hz for comparison. These frequencies were specifically chosen as they are common sampling frequencies for clinical ECG machines^32^ and have been shown to be good for heart rate variability analysis.^33^ These frequencies are a quarter, half, and three-fourths of the original sampling frequency, and thus this allows simple resampling without the need for interpolation of data points. Upsampling was not performed to avoid any possibility of adjusting the morphology of the signals before analysis; as the data were originally sampled at 500 Hz, this was taken as the maximum sampling frequency for this work.

Machine learning was performed using K-nearest neighbors (KNN) and support vector machine (SVM), considering the complexity value for each of the 12 leads individually and by creating a feature table with the complexity value for all 12 leads simultaneously and analyzing the combined feature table. Bayesian optimization was used to find the optimal values of the hyperparameters, taking account of the number of neighbors (all odd values from 1 to 99 to avoid tiebreaks) and distance function (10 options) for KNN and kernel type (linear and quadratic polynomials and Gaussian kernel) and outlier fraction (from 0 to 0.5) for SVM. The optimization works by aiming to minimize the output of a specific function; in this case, the function output was taken as 1 – accuracy. Alternatively, the output variable could have been determined using the area under the receiver operating characteristic curve, being defined as 1 – area under the curve. However, it was discovered that this caused issues in the optimization because of taking thresholds across a single value and so was later removed. When running a KNN machine learning model, 300 iterations of optimization were taken to encompass a wide range of the total 500 combinations of neighbors and distance metric without needing to evaluate all combinations. As the SVM models had a substantially smaller number of combinations, only 50 iterations were taken to ensure shorter computing time. Early results showed poorer results using SVM models, so KNN was used for the remainder of the results.

To ensure that the performance of the machine learning models was never evaluated using data that it had already seen, the leave-one-out cross-validation method was used. All records from a single patient were removed and tested using a machine learning model trained on the remainder of the training set to provide a classification. These classifications were recorded for all patients. Accuracy was determined by the percentage of total correct classifications. This cross-validation process was repeated for each iteration during optimization to ensure a fair validation of each combination of parameters. It is common practice to use hold out data sets; however, because of the size of the data set available for this work, it was determined that the leave-one-out method was preferable to avoid any underfitting resulting from the exclusion of training data.

To determine the optimal hyperparameters for each lead, coarse graining technique, and complexity algorithm, all combinations of optimal hyperparameters with the highest accuracy were taken. In some cases, there was only 1 set of hyperparameters. If there were multiple sets of hyperparameters all giving the optimal performance, then the mode of each individual hyperparameter value was taken. It was then checked that this combination of hyperparameters was one of the optimal options and there were no situations where the combination was not a previously checked option. Including the possibility of all 12 leads individually, all 12 leads combined, and all possible combinations of coarse graining and complexity methods results in a total of 1248 cases.

## Results

After optimizing all parameters across lead, coarse graining, and complexity algorithm combinations, the accuracy for each optimal combination was recorded.

**Figure 1:**
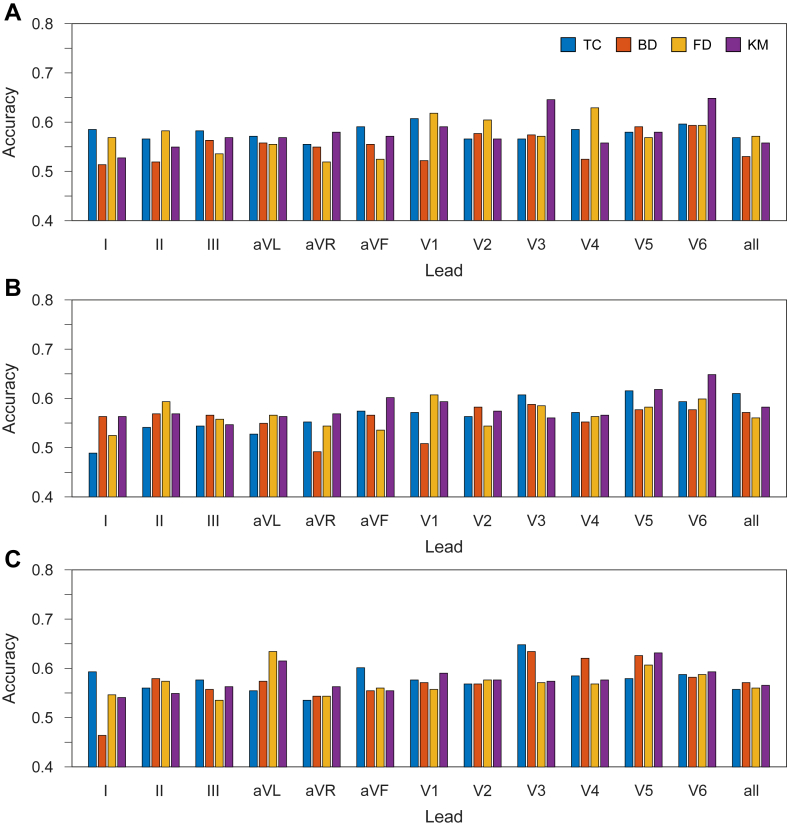
Accuracies across all 12 leads, including all leads combined, at 500 Hz using all coarse graining techniques: (**A**) for LZ76, (**B**) for LZ78, (**C**) for Titchener. BD = Beat Detection; FD = Feature Detection; KM = K-means; TC = Threshold Crossing.

Figure 1 shows the accuracy results for the 3 complexity algorithms with each coarse graining technique when the data are sampled at 500 Hz. The results for all other sampling frequencies were similar, and this figure helps motivate the choice of average as the median because of the low range of values across all leads. For the LZ76 results, the maximum accuracy is 0.646 (lead V_3_, KM). While some outlier minima are present, a majority of which occur for BD methods, most results occurred at the 0.6 mark. This can be seen to be the same for both the LZ78 and Titchener plots, with both having a maximum accuracy of 0.648 (LZ78: lead V_6_, KM; Titchener: lead V_3_, TC), and most other results lying around 0.6 accuracy. It can be expected that the accuracy for BD would be independent of the lead, as the R-wave peak will exist in the same place irrespective of which lead is being considered. It is likely that the different parameters for each lead, in conjunction with the training parameters from the machine learning model, create the change in accuracies.

LZ76 and LZ78 showed low variation across the BD values, with LZ76 achieving a maximum of 0.593 and a minimum of 0.514. LZ78 achieved a maximum of 0.582 and a minimum of 0.552; these give ranges of 0.079 and 0.03, respectively. Titchener showed a higher variation with a maximum accuracy of 0.635 and a minimum accuracy of 0.464; however, this appears to be an outlier and the lowest remaining accuracy is 0.544, giving a range of 0.091. While this is not vastly different from LZ76, it does show a wider range of values and the existence of the outlier amplifies this further.

**Figure 2:**
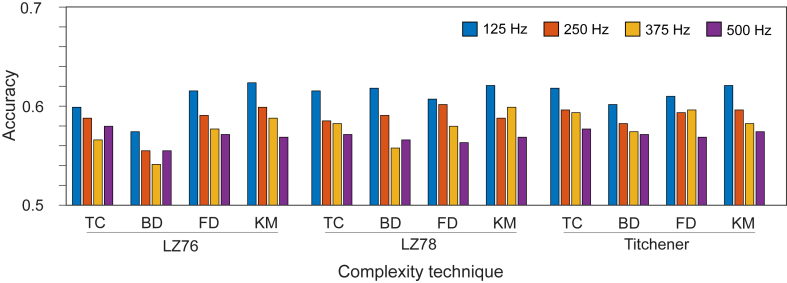
Comparison of accuracies at different sampling frequencies for each individual combination of coarse graining and complexity methods, calculating the accuracy using the median value across all leads. Abbreviations as in Figure 1.

Some of the occurring minima are well below the average accuracy, and these outliers could drastically affect the overall accuracy of each combination. Therefore, the median value across all leads was taken for each sampling frequency and individual combination of coarse graining and complexity methods, as can be seen in Figure 2.

While the range of accuracies across each individual combination of coarse graining and complexity methods is small, the overall pattern of an increased accuracy from a lower sampling frequency is clear. The maximum value of 0.624 was achieved at 125 Hz for LZ76(KM), with LZ78(KM) and LZ78(BD) obtaining a similar accuracy.

Table 2 shows that a majority of the top 10 most accurate combinations are generated from 125 Hz data. The minimum accuracy from these results was 0.462 (Ti(FD), lead I with 81 neighbors and Chebyshev distance metric), giving a range of 0.228.

**Table 2:** Top 10 most accurate methods along with the associated lead, sampling frequency, accuracy, sensitivity, and specificity

Algorithm	Lead	Sampling frequency	Accuracy	Sensitivity	Specificity
LZ78 (TC)	V_6_	125	0.690	0.582	0.797
Ti (TC)	V_6_	125	0.690	0.599	0.780
LZ78 (FD)	V_6_	125	0.679	0.615	0.742
Ti (KM)	V_5_	250	0.676	0.615	0.736
LZ76 (KM)	V_5_	250	0.673	0.555	0.791
LZ78 (KM)	V_5_	375	0.668	0.533	0.802
LZ78 (KM)	V_5_	250	0.662	0.544	0.780
Ti (BD)	V_6_	125	0.662	0.599	0.725
Ti (FD)	V_5_	125	0.662	0.533	0.791
LZ78 (BD)	All	375	0.659	0.500	0.819

BD = Beat Detection; FD = Feature Detection; KM = K-means; TC = Threshold Crossing.

Table 2 contains information on the hyperparameters and the sensitivity and specificity of each combination. PAF was taken to be the positive class, so sensitivity gives an indication of the model’s ability to predict patients with PAF. Even with an accuracy of at least 0.65, these models showed a poor performance, only slightly better than chance (50% accuracy). Instead, these models are better at determining control patients, with ∼80% of control patients being correctly identified. This could still be useful because in the real world a majority of patients will be controls, meaning this method could see viability as a screening test.

**Figure 3:**
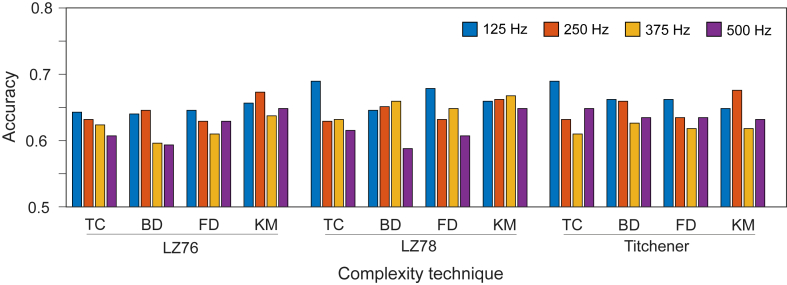
Comparison of accuracies at different sampling frequencies for each individual combination of coarse graining and complexity methods. The accuracy was taken as the maximum value across all leads for each individual combination of coarse graining and complexity methods. Abbreviations as in Figure 1.

Figure 3 shows a comparison of accuracies for each individual combination of coarse graining and complexity methods, this time choosing the maximum value across all leads rather than the mean. Although there is no longer a distinct pattern, 125 Hz still performs well compared with other sampling frequencies including having the highest 3 accuracies of 0.690 for LZ78(TC) and Ti(TC) and 0.679 for LZ78(FD). The sampling frequencies 375 and 500 Hz perform worst a majority of the time. The cases where 250 Hz outperforms 125 Hz are exclusively when KM is used as the coarse graining technique, and so it is likely that this method favors 250 Hz.

**Figure 4:**
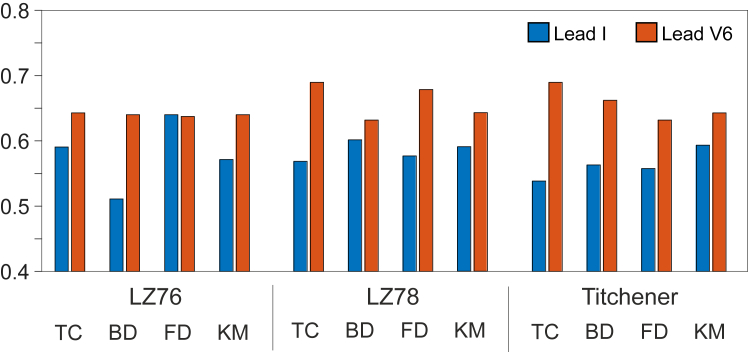
Comparison of accuracies between lead I and lead V_6_ for all coarse graining and complexity method combinations at 125 Hz. Abbreviations as in Figure 1.

**Figure 5:**
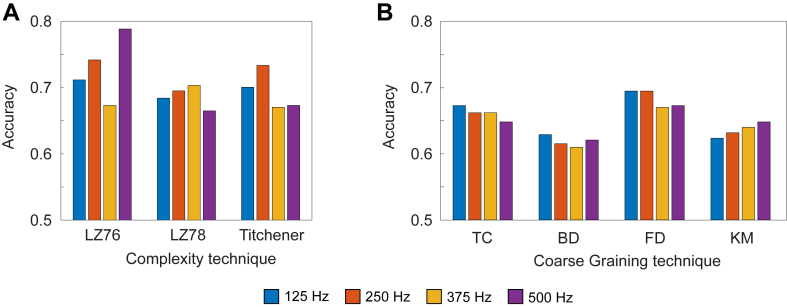
Comparison of maximum accuracies across sampling frequencies. **A:** Each section represents using all coarse graining methods for a single complexity method. **B:** Each section represents all complexity methods for a single coarse graining method.

**Figure 6:**
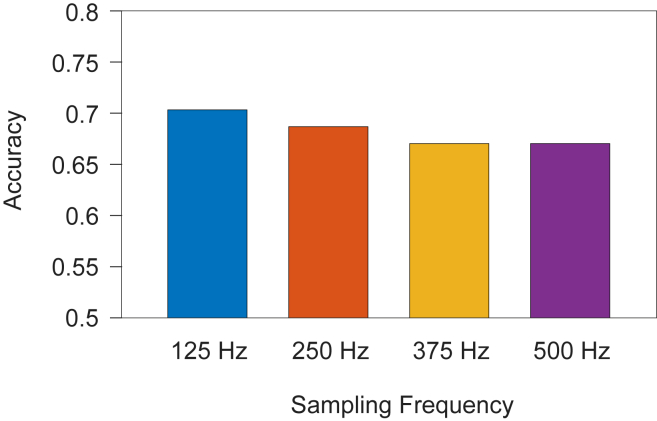
Comparison of maximum accuracies across sampling frequencies when using all coarse graining and complexity algorithm combinations simultaneously.

Figure 4 shows a comparison of accuracies between lead I and lead V_6_ for each individual combination of coarse graining and complexity methods, showcasing the predictive power of each lead. Lead I obtained a maximum of 0.640 accuracy (LZ76(FD)) with a mean accuracy of 0.555, whereas lead V_6_ obtained a maximum of 0.690 accuracy (LZ78(TC) and Ti(TC)) with a mean accuracy of 0.617. Figure 5 considers maximum accuracies for all sampling frequencies across all leads, including all leads simultaneously. Figure 5A shows the results when the feature table used a single complexity method with all available coarse graining techniques, that is, LZ76(TC), LZ76(BD), LZ76(FD), and LZ76(KM) combined, with the same for other complexity methods. The maximum value was achieved for LZ76 at 500 Hz with 0.788 accuracy, while at 125 Hz it had an accuracy of 0.712. Figure 5B shows the results when the feature table used a single coarse graining technique with all available complexity methods, that is, LZ76(TC), LZ78(TC), and Ti(TC), with the same for other coarse graining techniques. FD has the highest accuracy of 0.695, while KM achieved a highest accuracy of 0.648. Figure 6 shows maximum accuracies across all sampling frequencies when the complexity scores across all coarse graining and complexity methods were used in a single feature table; that is, all 12 complexity scores were used simultaneously. The sampling frequency 125 Hz has an accuracy of 0.703, and 500 Hz has an accuracy of 0.670.

As discussed, the prevalence of AF is dependent on many factors, including, but not limited to, age and sex. For this reason, we investigated the impact of including the age and sex of patients as part of the feature table provided to the machine learning model and the impact on accuracy for each of the best results previously presented. First, we consider the best accuracy from Figure 3, LZ78(TC) at 125 Hz with lead V_6_. We saw this had accuracy of 0.690 but now with the same parameters and the addition of the age and sex features, the accuracy decreases to 0.635. Second, in Figure 5A, the LZ76 features with all coarse graining techniques achieved an accuracy of 0.789 with a 500-Hz sampling frequency; however, the accuracy remained at 0.789 with the addition of the age and sex features. Third, in Figure 5B, the optimal accuracy of 0.695 was achieved using FD coarse graining with all complexity methods at 125 Hz. This showed a slight improvement to 0.696 with the addition of age and sex features. Finally, the use of all coarse graining and complexity method features yielded an accuracy of 0.703 at 125 Hz, as can be seen in Figure 6. With the addition of the age and sex features, this increased to 0.707.

## Discussion

In this study, our principal findings are as follows:

1. 125 Hz is the optimal sampling frequency for the complexity analysis of 10-second ECGs for a diagnosis of AF from patients currently in SR.
2. Leads V_5_ and V_6_ provide the highest accuracy when using a KNN model.

### Previous analysis

In 2019, a retrospective study explored whether AI can detect AF using ECGs recorded in the 31 days before a known AF event.^14^ Using a total of 649,931 records (12-lead, 10-second ECGs recorded at 500 Hz), the convolutional neural network achieved an accuracy of 79%–83% with the sensitivity and specificity existing in the same range and differing by <1%; this means that the model is able to predict both cases (PAF) and controls (healthy) with equal/the same ability. A limitation is a lack of discussion of the type of AF of the patients. The size of the data set is considerably larger than most, and all 12 leads were available, as machine learning models by nature are dependent on large amounts of data for learning. Although the success of this model will not be entirely determined by the size of the data set, AI models can perform optimally when presented with a large amount of data.

AI learning methods work across a range of techniques, and understanding how changes in the data set can influence the sensitivity and specificity of the results is important. Alexeenko et al^34^ used complexity algorithms with 28-second, single-lead, 125-Hz recordings taken from 57 patients. By using metrics from these complexity algorithms in conjunction with a classification learner, this model was able to achieve a sensitivity of 89% and a specificity of 83%.

A direct comparison of performance metrics from these models shows that changes in methodology can affect sensitivity by up to 10%. Most significantly, sampling frequency is an important determining factor. It determines how many readings a second the ECG monitor records; a 10-second recording at 500 Hz would contain 5000 data points. Recording at a higher sampling frequency naturally allows more data to be recorded, and machine learning models provide more accurate predictions with more data,^35^ as it reduces the possibility of overfitting. Sampling frequency does not always affect the amount of information provided to a machine learning model, and the study by Alexeenko et al^34^ reinforces the need to understand the optimal data set parameters for analysis.

Complexity has previously been used to diagnose PAF from ECGs in SR.^15^^,^^36^ The lack of formalization regarding the optimization and impact of sampling frequency makes reproducibility and future work using complexity difficult. Therefore, we progressed to formalize the impact of sampling frequency when using complexity analysis to discriminate between patients with PAF and healthy patients exhibiting ECGs in SR.

### Methods-based discussion

Models tend to perform better at lower sampling frequencies for several reasons. With higher frequencies the impact of any noise is higher, particularly when using TC and KM, where more points are being included in the binary string. This could lead to more variability in the complexity values, making it harder for the machine learning models to discriminate between cases and controls. Another possibility is that adding more points to the binary string artificially raises the complexity value toward the upper limit. For BD and FD coarse graining techniques, the relative position of the detected features is unchanged for different sampling frequencies—all that changes is the number of zeros between each one, representing a feature in the signal. For an algorithm such as LZ78, it follows that this naturally increases the complexity value as more words of increasing longer sequences of zeros must be added. There is a known upper limit for the complexity value of a string, and so increasing the complexity value through sampling frequency could reduce the range of values each record can take; however, this may be compensated for by normalization.

We can also consider the (mis)labeling of patients in the original data set, as control patients may have undetected PAF episodes. An investigation into misclassified records showed that there were 5 records across 5 separate patients who were labeled as having PAF by the model in at least 9 of the 12 methods. Unfortunately, there is no way to follow up to see whether these patients later received a PAF diagnosis.

Algorithms including BD generally perform worse. The accuracy is lower for most frequencies, and FD performs similarly to other coarse graining techniques. This perhaps indicates that BD discards too much information necessary for the detection of PAF from SR signals. Thus, a change in R-R intervals alone is not sufficient to detect PAF. It would be beneficial to examine the impact of different choices of features in FD or different thresholds for TC, allowing a different range of features to be encompassed as part of coarse graining.

More supporting evidence toward this is the appearance of KM 4 times in Table 2 in comparison to BD appearing once. In a way similar to TC, KM retains more information about the signal as it will likely include some information on P and T waves. Although KM is susceptible to baseline wander and noise like TC, the results show that it must be able to more accurately distinguish between case and control patients. It follows that more work could be done in the future to investigate the impact of different signal features with regard to the diagnosis of PAF.

### Results-based discussion

An interesting result from Table 2 is the high prevalence of the chest leads V_5_ and V_6_, appearing a combined 9 times out of the top 10 results. Figure 4 shows the comparative accuracies for leads I and V_6_ at 125 Hz for all coarse graining and complexity method combinations. While the results are similar, there is a clear difference in both maximum achieved accuracy and average accuracy. This 5% increase in both maximum value and mean value for lead V_6_ shows that this chest lead gives noticeable improvement compared with the remaining leads.

The frequency 125 Hz is the most common sampling frequency, appearing 5 out of the 10 times in Table 2, with 250 Hz appearing 3 times. The frequency 375 Hz does appear 2 times; however, it is likely that these good results are outweighed by the other considerably worse accuracy values obtained for other results when using 375Hz sampling frequency, giving the overall trend seen in Figure 2. The frequency 500 Hz does not appear at all, an unsurprising result considering it is often the worst-performing sampling frequency across the individual coarse graining and complexity method combinations. While this also factors in the choice of distance metric and number of neighbors for the machine learning model, it shows the sensitivity of machine learning models to their input parameters, with sampling frequency being the most prevalent for this model.

All coarse graining and complexity methods appear in the top 10; LZ78 and Ti are the most common of the complexity methods appearing 5 and 4 times, respectively. The coarse graining techniques contain a slightly more even split, with KM appearing 4 times and all other methods appearing 2 times. There was little repetition across these combinations, which likely means that the individual methods themselves do not have a large impact on the results, but rather certain combinations show slight improvement over others. This matches with the findings in Figure 2.

Figure 5 shows a comparison of the maximum accuracies when the feature tables are taken using individual combinations of coarse graining methods and complexity methods. Figure 5A uses all coarse graining methods for a single complexity method, while Figure 5B uses all complexity algorithms for a single coarse graining method. Figure 5B follows a previous analysis with 125 Hz exceeding the accuracy of other sampling frequencies in most cases. For KM, we see that the pattern is actually reversed, with 500 Hz performing best, and for FD, 250 Hz has an accuracy equal to that of 125 Hz.

Figure 5A shows less correlation with the results appearing unrelated to their sampling frequency. LZ78 and Ti showed more stable results, but the maximum values were achieved for 375 and 250 Hz, respectively. Thus, the coarse graining techniques, but not complexity methods, were affected by sampling frequency.

Figure 6 shows the maximum accuracy when the feature table was taken as all 12 individual combinations of coarse graining techniques and complexity algorithms. These results return to the original pattern seen with 125 Hz performing best and a consistent decrease in accuracy down to 500 Hz. The range of accuracies is small; however, it still holds that 125 Hz performs best for all features combined. Sampling frequency affects the accuracy across leads and coarse graining techniques but has less impact on the complexity methods themselves.

The investigation into the impact of age and sex predictors showed slight improvement in all cases except for a single complexity score. Because of the nature of the matched data set created in the Data Set Breakdown section, it is likely that age and sex play a less significant role than they do when considering the general population. If the spread of age and sex in the database was indicative of that of the general population, then the prevalence of AF in older or male patients would potentially increase the predictive power of age and sex features. As the database was designed with the age and sex of control patients to match patients with AF, it sees less impact and, in the case of a single complexity score, hinders the predictive power, likely because of the increased spread of the data. It is possible that when using more complexity score features, the impact the age and sex features have on the model is less significant and therefore has little to no impact on accuracy.

Although the difference between all sampling frequencies is low across all methods and combinations, we need to consider both the size of our data set and the impact these changes would have on a larger population. As this is a new area of research, the number of databases with appropriate recordings of high quality is low; most AF databases look to include the AF episode as part of the ECG recording to aid in episode detection. Instead we are interested in the section of ECG recording following the AF episode (as outlined in the Data Set Breakdown section) which is sometimes omitted. As a result, this lead to the study being performed with a fairly small database. We note however that 5% increase in accuracy could lead to a drastic increase in the number of patients being correctly diagnosed in a larger database.

## Comparison and future work

Upon comparing these results with those from Alexeenko et al,^34^ it is encouraging to note that 125 Hz was considered the optimal sampling frequency from this analysis and was the sampling frequency previously used. While the specificity values are similar, there is a large disparity between the sensitivity values of the 2 studies: Alexeenko et al was much better at diagnosing PAF. One option is that the machine learning model should have been expanded to include the results of the “h score”, a metric designed to measure the variability of different complexity scores from the same ECG used by Alexeenko et at. These findings contrast with those from Attia et al,^14^ which achieved similar results using a machine learning tool using ECG signals alone. Alternatively, the length of the records used was a limiting factor in the results. Future work should look to evaluate the impact of strip length with complexity analysis and fully elucidate the findings from this study with an application to a larger data set.
