# Supplementary material for: Electrocardiogram sampling frequency for the optimal performance of complexity analysis and machine learning models: Discrimination between patients with and without paroxysmal atrial fibrillation using sinus rhythm electrocardiograms
Source: Heart Rhythm O2. 2024 Nov 8;6(1):48–57. doi: 10.1016/j.hroo.2024.11.002 (PMC11993800; doi:10.1016/j.hroo.2024.11.002)
Supplement: Supplementary Appendix [file mmc1.docx]

# Supplementary Appendix

*Electrocardiogram sampling frequency for optimal performance of complexity analysis and machine learning models: Discrimination between subjects with and without paroxysmal atrial fibrillation using sinus rhythm electrocardiograms*

## Historical aspects and technical details of the complexity algorithms: LZ76, LZ78 and Titchener methods

Early work by Kolmogorov [1] paved the way for programming that would be able to estimate the complexity of a finite string. A large part of this was done by Lempel and Ziv in 1976 [2] and later revised in 1978 [3,4], hence the naming of the methods as LZ76 and LZ78. Titchener [5] later developed another variation (which we will refer to as Ti) to avoid incomplete parsing.

The LZ76 algorithm determines the complexity of a finite string by the number of distinct ‘components’ (defined later) in the string. Consider two strings S and Q, where Q is known as an extension of S, and define R=SQ. Initially, S is empty and Q is the first symbol in the string. The extension Q is ‘reproducible’ from S if Q is a substring of R without the final symbol. If we were to include the entirety of R, Q would trivially be a substring, but by excluding the final symbol it guarantees that the substring will start at some point in S, and will possibly, but not necessarily, including some symbols from Q. Let S’ and Q’ be the strings at the next step of the process. If the extension Q is reproducible, we take S’=S and define Q’ to be Q with the addition of the next symbol in the string. Thus, S stays the same but we add a symbol to Q. If Q is not reproducible, it is taken as a new component and in this case, S’=R=SQ, and therefore becomes the whole of the current string being considered, and Q’ is the next symbol in the string. We then checked whether Q’ is reproducible from S’. This process continues until the end of the original string is reached and the number of distinct components found is the complexity [2].

LZ78 looks to generate a dictionary of patterns within the string, which are known as ‘words’. Again, consider R=SQ where Q is an extension of S but this time the extension Q is compared with the words saved in the dictionary. If Q does not match any of the previously saved words then Q is added to the dictionary as a new word and S’=R=SQ and Q’ is the next symbol in the string. Similarly if Q does match a word in the dictionary then S’=S and Q’ is Q with the addition of the next symbol in the string. It therefore follows that the dictionary will be unique substrings of S that exactly partition S. The complexity of the full string is given by the number of words in the dictionary when the whole string has been processed in this way [4]. The difference between LZ76 and LZ78 is that for LZ78 Q must be one of the unique words of S whereas for LZ76 Q must be a substring of the full string R, excluding the final symbol.

The Titchener (Ti) method was motivated by LZ76 and LZ78 where the last extension Q is reproducible/already in the dictionary but there are no more symbols to add. This creates a problem as to whether this last extension should increase the complexity value by one or not [6]. Instead of processing the string from beginning to end as in the other methods, the Titchener method begins by partitioning the entire signal into individual symbols and the algorithm joins partitions together systematically. The starting alphabet is considered to be {0,1} and the full string is partitioned such that every partition is an element of this alphabet. An arbitrary symbol “a” is appended to the signal such that the last symbol of the string is now the penultimate partition. The penultimate partition of the string is taken as a starting point and the full string is checked for copies of this partition. The penultimate partition is used to update the alphabet by taking copies of the other elements of the alphabet, the process repeats until the alphabet contains the entire string as an element. The complexity value is taken as the sum of log_2_(k+1) where k is the number of copies of the penultimate partition at each step of the algorithm. The full algorithm can be found in Clark and Teutsch [5].

## References

[1] A.N. Kolmogorov, Three approaches to the quantitative definition of information, Int J Comput Math 2 (1968) 157–168. https://doi.org/10.1080/00207166808803030.

[2] A. Lempel, J. Ziv, On the Complexity of Finite Sequences, IEEE Trans Inf Theory 22 (1976) 75–81. https://doi.org/10.1109/TIT.1976.1055501.

[3] J. Ziv, A. Lempel, Compression of individual sequences via variable-rate coding, IEEE Trans Inf Theory 24 (1978) 530–536. https://doi.org/10.1109/TIT.1978.1055934.

[4] J. Arz, J. Fischer, Lempel–Ziv-78 Compressed String Dictionaries, Algorithmica 80 (2018) 2012–2047. https://doi.org/10.1007/s00453-017-0348-7.

[5] G. Clark, J. Teutsch, Maximizing T-complexity, Fundam Inform 139 (2015) 1–19. https://doi.org/10.3233/FI-2015-1223.

[6] U. Speidel, A note on the estimation of string complexity for short strings, in: 2009 7th International Conference on Information, Communications and Signal Processing (ICICS), IEEE, 2009: pp. 1–5. https://doi.org/10.1109/ICICS.2009.5397536.
